# Adjuvanted Recombinant Hemagglutinin Vaccine Provides Durable and Broad-Spectrum Immunogenicity in Mice

**DOI:** 10.3390/vaccines13111162

**Published:** 2025-11-14

**Authors:** Rui Yu, Yan Guo, Senyan Zhang, Yuanbao Ai, Rui Wei, Yan Li, Hang Chen, Shuyun Liu, Caixia Zhang, Yuanfeng Yao, Meng Lv, Yingying Li, Yulin Chen, Peng Zhou, Siting Tu, Meijuan Fu, Yongshun Su, Yu Lin, Min Yang, Yanbin Ding, Siyu Tian, Cai Jing, Hang Chen, Tao Ma, Chunping Deng, Yu Zhou, Yuanyuan Li, Jing Jin

**Affiliations:** Patronus Biotech Co., Ltd., Guangzhou 510005, China; yurui@luye.com (R.Y.); guoyan02@luye.com (Y.G.); senyanzhang@gmail.com (S.Z.); smileday530@163.com (Y.A.); weirui@luye.com (R.W.); liyan06@luye.com (Y.L.); chenhang01@luye.com (H.C.); liushuyun@luye.com (S.L.); zhangcaixia01@luye.com (C.Z.); yaoyuanfeng@luye.com (Y.Y.); lumeng@luye.com (M.L.); liyingying@luye.com (Y.L.); chenyulin@luye.com (Y.C.); zhoupeng01@luye.com (P.Z.); tusiting@luye.com (S.T.); fumeijuan@luye.com (M.F.); suyongshun@luye.com (Y.S.); linyu02@luye.com (Y.L.); yangmin02@luye.com (M.Y.); dingyanbin@luye.com (Y.D.); tiansiyu@luye.com (S.T.); jingcai@luye.com (C.J.); chenhang@luye.com (H.C.); matao@luye.com (T.M.); dengchunping@luye.com (C.D.); zhouyu01@luye.com (Y.Z.)

**Keywords:** recombinant influenza vaccine, hemagglutinin, durability, broad-spectrum

## Abstract

**Background**: Seasonal influenza vaccines must be reformulated annually due to the high genetic variability and antigenic drift of circulating influenza viruses. The annual update, guided by World Health Organization (WHO) recommendations, results in significant challenges, including compressed production time periods, elevated manufacturing costs, and global distribution pressures. Moreover, mismatches between vaccine strains and circulating viruses can severely reduce protective efficacy, underscoring the urgent need for broadly protective and long-lasting influenza vaccines. **Methods**: In this study, we developed an adjuvanted trivalent recombinant influenza virus-like particle vaccine (a-RIV) using the baculovirus–insect cell expression system and formulated it with an AS01-like adjuvant. The vaccine comprises full-length hemagglutinin (HA) proteins from WHO-recommended seasonal influenza strains: A/H1N1 (AH1), A/H3N2 (AH3), and B/Victoria (B/vic) lineages. The purified HA proteins were subsequently formulated with a liposomal adjuvant to enhance the immunogenicity. **Results**: In mouse immunization studies, the a-RIV vaccine elicited significantly stronger humoral and cellular immune responses than the licensed recombinant vaccine Flublok and the conventional inactivated influenza vaccine (IIV). High levels of functional anti-HA antibodies and antigen-specific T cell responses persisted for at least six months post-vaccination. Moreover, a-RIV induced broadly reactive antibodies capable of cross-binding to heterologous AH1 and AH3 influenza strains. **Conclusions**: Our data demonstrate that the a-RIV elicits enhanced, durable, and broadly cross-reactive immune responses against multiple influenza subtypes. These findings support the potential of adjuvanted recombinant HA-based vaccine as a promising candidate for the development of next-generation influenza vaccine.

## 1. Introduction

Influenza is a leading cause of lower respiratory tract infections (LRTIs) across all age groups, accounting for approximately 11.5% of all LRTI cases globally. In 2017 alone, the influenza virus was responsible for an estimated 54.5 million LRTI episodes, including 8.17 million severe cases worldwide [1]. Beyond LRTIs, influenza contributes to a broader spectrum of disease burden, including cardiovascular events, exacerbation of chronic diseases, secondary bacterial infections, functional decline, and adverse pregnancy outcomes, especially among immunocompromised individuals [2]. These complications significantly increase the risk of hospitalization and mortality, with approximately 145,000 influenza-related deaths reported globally each year [1].

Vaccination remains the most effective strategy for preventing influenza infection. Current vaccine platforms include inactivated vaccines (whole-virus, split-virus, and subunit), live-attenuated vaccines, and recombinant protein vaccines [3]. Among them, egg-based inactivated and live-attenuated vaccines are widely used but are unsuitable for individuals with egg allergies and may conflict with religious dietary restrictions [3]. In contrast, recombinant vaccines such as Cadiflu-S [4] and Flublok (also marketed as Supemtek Tetra) [5] eliminate egg-derived components and enable faster, more scalable production. However, a key limitation of currently licensed recombinant influenza vaccines is the lack of an adjuvant.

Adjuvants have been shown to significantly enhance the immunogenicity of inactivated influenza vaccines, particularly in populations with weaker immune responses. For example, MF59-adjuvanted vaccines are widely approved for use in older adults aged ≥65 years to reduce the risk of severe influenza and its complications [6,7] and are also approved for children ≥6 months old in several countries, including Canada [8]. Despite these benefits, no adjuvanted recombinant influenza vaccines are currently available.

Meanwhile, the effectiveness of seasonal influenza vaccines remains highly variable. Between the 2009–2010 pandemic and the 2019–2020 flu seasons, the overall vaccine effectiveness (VE) was moderate in the Southern Hemisphere (54%; 95% CI: 48–59%) but lower in the Northern Hemisphere (37%; 95% CI: 32–42%) [9]. According to data from the U.S. Centers for Disease Control and Prevention (CDC), VE between 2009 and 2025 ranged from 19% to 60%. The lowest VE (19%) occurred during the 2014–2015 season, primarily due to antigenic drift in the circulating A/H3N2 strain that led to a mismatch with the vaccine strain [10]. This antigenic drift is primarily driven by the lack of proofreading capability in the viral RNA-dependent RNA polymerase, leading to a high mutation rate [11,12]. Among the major influenza subtypes, A/H3N2 evolves the fastest, with an estimated adaptation rate of 5.7 × 10^−3^ substitutions per codon per year, followed by A/H1N1 (3.2 × 10^−3^) and the B/Victoria lineage (1.8 × 10^−3^).

Taken together, these limitations underscore the urgent need for next-generation influenza vaccines that combine recombinant production platforms with potent adjuvants to enhance immunogenicity and provide broader, more durable protection against rapidly evolving influenza viruses. In this context, structural insights into influenza virions become particularly important. The viral envelope is densely packed with surface glycoproteins—approximately 300–400 HA and 40–50 neuraminidase (NA) molecules per 120 nm virion [13]. Among these, HA plays a crucial role in mediating viral attachment to host sialic acid receptors [14] and remains the primary antigenic target in influenza vaccine development [15].

In this study, we developed a trivalent adjuvanted recombinant influenza virus-like particle vaccine (the a-RIV vaccine) based on AH1, AH3, and B/vic HA antigens and subsequently evaluated its immunogenicity, durability, and cross-reactivity in mice to assess its potential as a next-generation influenza vaccine candidate.

## 2. Materials and Methods

### 2.1. Cells and Virus

Sf9 cells were purchased from Gibco (Grand Island, NY, USA) and cultured in ExpiSf^TM^ CD Medium, cells were maintained in shake flasks on a shaker platform in a non-humidified, non-CO_2_ cell culture incubator at 27 °C. Huh-7 and MDCK cells were maintained in Dulbecco’s Modified Eagle Medium (DMEM; Gibco) supplemented with 10% fetal bovine serum (FBS; Gibco) and 1% penicillin–streptomycin (Gibco), and cells were cultured at 37 °C in a humidified incubator with 5% CO_2_. Chicken and guinea pig red blood cells (RBCs) were obtained from SenBeiJia Biological Technology Co., Ltd. (Nanjing, China) and stored at 2–8 °C. Influenza virus strains were provided and preserved by the Changchun Institute of Biological Products Co., Ltd., (Changchun, China).

### 2.2. Construct Design and Protein Expression

The codon-optimized nucleotide sequences of full-length AH1 HA, AH3 HA, and B/vic HA, along with their secreted variants carrying a C-terminal Spytag, a foldon domain, and a His tag, were synthesized by GenScript (China). For the membrane-anchored HA (HA_mem_) antigens, the native signal peptide was replaced with the sequence MPLYKLLNVLWLVAVSNA, followed by the full-length HA amino acid sequence, including the ectodomain, transmembrane, and cytoplasmic domains. The expressed regions correspond to residues A17–I566 for A/Wisconsin/67/2022 (H1N1), Q17–I566 for A/Massachusetts/18/2022 (H3N2), and D16–L582 for B/Austria/1359417/2021 (B/Victoria). Retaining the transmembrane and cytoplasmic domains enables insertion of HA trimers into the insect cell membrane, where they associate through hydrophobic interactions and oligomerization. Upon detergent-mediated membrane disruption, these membrane-bound HA trimers self-associate to form nanoparticle-like aggregates (typically 20–40 nm in diameter, comprising approximately 5–20 trimers), mimicking the surface organization of influenza virions. For the secreted HA (HA_sec_) constructs, the transmembrane and cytoplasmic regions were removed, and the signal peptide was replaced with MVSAIVLYVLLAAAAHSAFA to facilitate secretion. The expressed ectodomains spanned A17–V520 (H1N1), Q17–V521 (H3N2), and D16–S535 (B/Victoria). Because these constructs lack the membrane anchor, the trimeric HA proteins are released into the culture supernatant upon expression and subsequently purified from the medium.

Each HA gene was subsequently cloned into the pOET5.1 transfer plasmid (OET, Oxford, UK). These recombinant plasmids were then co-transfected with flashBAC™ baculovirus genomic DNA (OET) into Sf9 cells using baculoFECTIN II transfection reagent (Mirus Bio, Madison, WI, USA). Following a 5-day incubation, the culture supernatants were harvested to collect the recombinant baculoviruses. These recombinant viruses were subsequently used to infect Sf9 cells at a multiplicity of infection (MOI) of 1. Infected cells were cultured at 27 °C for 3–4 days, after which they were harvested by centrifugation at 2000× *g* for 30 min. Expression of full-length HA led to the formation of HA membrane protein virus-like particle (HA_mem_-VLP), whereas secreted HA variant (HA_sec_) was released into the culture supernatant. For HA_mem_-VLPs, the supernatants were discarded and the cell pellets were collected for subsequent protein purification. For HA_sec_ proteins, the cell pellets were discarded, and the supernatants were used for downstream purification.

### 2.3. Protein Purification

Purification of AH3 HA_mem_-VLP: Following the harvest, Sf9 cells were washed with PBS and lysed using a 1% NP-9 solution to extract AH3 HA membrane protein. The resulting lysate was clarified by centrifugation, and the supernatant was subjected to initial purification using a Diamond MMC Mustang ion-exchange chromatography column (Bestchrom, Shanghai, China). The elution buffer consisted of 20 mM phosphate buffer (PB), 5% glycerol, 0.3% NP-9, 0.05% cysteine, and 120 mM NaCl at a pH of 6.5. The eluted AH3 HA protein was subsequently adjusted to pH 7.0 and further purified using a ceramic hydroxyapatite (CHT) chromatography column (NanoMicro, Suzhou, China). The CHT elution buffer comprised 40 mM PB, 5% glycerol, 0.05% polysorbate 20 (Tween-20), 0.05% cysteine, and 200 mM NaCl at pH 7.0. The final purified AH3 HA_mem_-VLP was buffer-exchanged into PBS using ultrafiltration tube (Merck, Shanghai, China) and stored at 4 °C.

Purification of AH1 HA_mem_-VLP: Sf9 cells were washed with PBS and lysed in 1% Triton X-100 to extract AH1 HA membrane protein. The clarified lysate was loaded onto a Diamond MMC Mustang ion-exchange chromatography column. The elution buffer contained 20 mM PB, 5% glycerol, 0.1% Triton X-100, and 160 mM NaCl (pH 6.5). Then the eluted sample was adjusted to pH 7.0 for further purification using a CHT chromatography column. Elution was performed using a linear salt gradient with 10 mM PB, 5% glycerol, 0.05% Tween-20, and 0–1000 mM NaCl at pH 7.4. The final purified AH1 HA_mem_-VLP was subjected to buffer exchange using ultrafiltration into PBS and subsequently stored at 4 °C.

Purification of B/vic HA_mem_-VLP: After washing Sf9 cells, the B/vic HA membrane protein was extracted using a 1% NP-9 solution. The clarified supernatant was initially purified with a Nanogel 50Q HC ion-exchange chromatography column (NanoMicro). The partially purified protein was adjusted to pH 6.5 before undergoing a second purification step using a CHT chromatography column. The CHT elution buffer consisted of 20 mM PB, 210 mM NaCl, 0.05% Tween-20, at pH 7.4. The purified B/vic HA_mem_-VLP was buffer-exchanged into PBS via ultrafiltration and stored at 4 °C.

Purification of AH3, AH1 and B/vic HA_sec_ Proteins and Preparation of HA_sec_ nanoparticles (HA_sec_-NPs): The cell culture supernatants were first filtered through a 0.22 μm membrane and then purified on an ÄKTA system using prepacked HisTrap Excel and Superdex 200 Increase 10/300 GL columns, with buffer exchange into 20 mM Tris-HCl, 150 mM NaCl, pH 7.4, to obtain highly purified AH3, AH1, and B/vic HA_sec_ proteins. These HA_sec_ proteins were individually mixed with mi3 at a fixed mess ratio (mess ratio of HA_sec_: mi3 = 1:6) and incubated for 24 h at 22 °C to prepare nanoparticles. The resulting mixtures were subsequently separated on a Superose 6 Increase 10/300 GL column to isolate HA_sec_-NPs from unbound HA_sec_ proteins. Finally, HA_sec_-NPs were stored at –80 °C. All columns and the ÄKTA system were purchased from Cytiva (Hauppauge, NY, USA).

As previously described [16,17], the mi3 scaffold is a self-assembling 60-mer nanoparticle derived from 2-keto-3-deoxy-6-phosphogluconate (KDPG) aldolase. To enable site-specific antigen display via the SpyTag/SpyCatcher isopeptide conjugation system, the Catcher domain was genetically fused to the mi3 subunit using a flexible linker. The Catcher-mi3 construct was cloned into the pET30a vector and expressed in *Escherichia coli* BL21 (DE3) cells cultured in a 5 L bioreactor. Following expression, the bacterial cells were lysed using an AH-PILOT high-pressure homogenizer (ATS Engineering Ltd., Cambridge, Ontario, Canada), and the clarified lysate was purified using a multi-step protocol based on Good Manufacturing Practice (GMP)–compliant procedures. This purification strategy ensured high yield and structural integrity of the assembled 60-mer nanoparticles.

HA_mem_-VLPs and HA_sec_-NPs were evaluated by transmission electron microscopy (TEM). The samples were adsorbed onto freshly glow-discharged carbon-coated copper grids (EMCN Co.) for 1 min at room temperature. The grids were negatively stained with 2% phosphotungstic acid (Rhawn, R128447) for 60 s, air-dried, and examined using a Hitachi HT7800 (Hitachi, Chiyoda-ku, Tokyo, Japan) transmission electron microscope operating at 80–120 kV

### 2.4. Preparation of AS01-like Adjuvant and Formulation of Adjuvanted Vaccine

A measured amount of cholesterol (Avanti Polar Lipids, Alabaster, AL, USA), 1,2-dioleoyl-sn-glycero-3-phosphocholine (DOPC; Avanti Polar Lipids, Alabaster, AL, USA), and monophosphoryl lipid A (MPL; Sigma-Aldrich, St. Louis, MO, USA) was dissolved in isopropanol (Sinopharm Chemical Reagent Co., Ltd., Shanghai, China) and evaporated under reduced pressure using a RE-52AA rotary evaporator (Yarong Biochemical Instrument Factory, Shanghai, China) to form a lipid film. The lipid film was subsequently hydrated with sterile Milli-Q water (Millipore, Billerica, MA, USA) to generate a coarse liposome suspension, which was then processed with an Avestin EmulsiFlex-C3 high-pressure homogenizer (Avestin, Ottawa, Ontario, Canada) to reduce particle size and produce homogeneous liposomes. QS-21 (Desert King, San Diego, CA, USA) was then added to the liposome suspension to obtain the final adjuvant formulation, designated AS01-like adjuvant. The final composition of AS01-like adjuvant consisted of 4 mg/mL DOPC, 1 mg/mL cholesterol, and 0.2 mg/mL each of QS-21 and MPL.

For vaccine formulation, AS01-like adjuvant was mixed at equal volume with a trivalent recombinant HA protein preparation (comprising AH1, AH3, and B/vic antigens). Mice were immunized intramuscularly with 100 μL of the adjuvanted vaccine.

### 2.5. Animal Assay

Female BALB/*c* mice (6–8 weeks old) were purchased from Beijing Vital River Laboratory Animal Technology Co., Ltd. (Beijing, China), and housed at Forevergen Biotechnology Co., Ltd. (Guangzhou, China), where all animal experiments were conducted. All procedures were approved by the Institutional Animal Care and Use Committee (IACUC) of Forevergen Biotechnology (approval No. IACUC-AEWC-F2401021) and were performed in strict accordance with the 3Rs principles (Replacement, Reduction, and Refinement).

### 2.6. Enzyme-Linked Immunosorbent Assay (ELISA)

**Figure 1:**
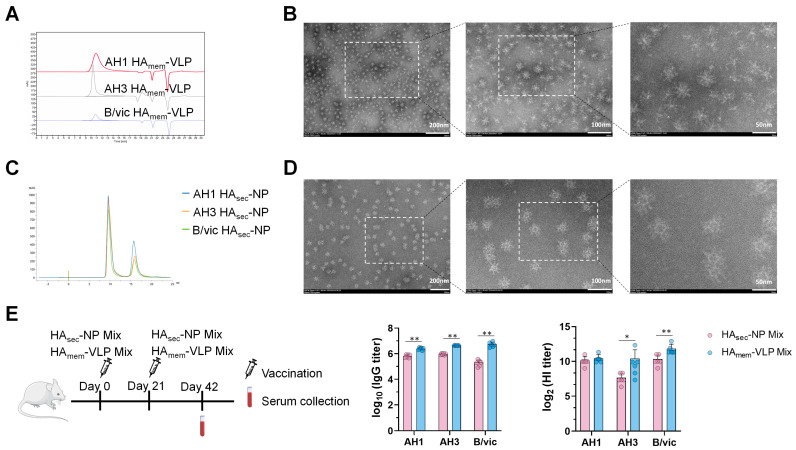
Characterization of HA_mem_-VLPs, HA_sec_-NPs and immunogenicity. (**A**) Size-exclusion chromatography profiles of purified HA_mem_-VLPs, indicating absorbance (Y-axis) and retention time (X-axis), AH1, AH3, and B/vic are shown in red, black, and purple, respectively. Negative-stain electron microscopy images of the mixed AH1, AH3, and B/vic HA_mem_-VLP mix (**B**) and HA_sec_-NP mix (**D**). Left, middle, and right panels correspond to magnifications of 50,000× (**B**) or 40,000× (**D**), 100,000×, and 200,000×, respectively. (**C**) SEC profiles of purified HA_sec_-NPs, indicating absorbance (Y-axis) and retention volume (X-axis), AH1 (blue), AH3 (orange), and B/vic (green). (**E**) Mice were immunized with the adjuvanted HA_sec_-NP mix and HA_mem_-VLP mix on day 0 (prime) and day 21 (boost), and sera were collected on day 42. Antigen-specific IgG titers (left) and HI titers (right) were measured (n = 6). Statistical significance was determined using an unpaired *t*-test (* *p* ≤ 0.05, ** *p* ≤ 0.01). Non-significant difference is not shown.

**Figure 2:**
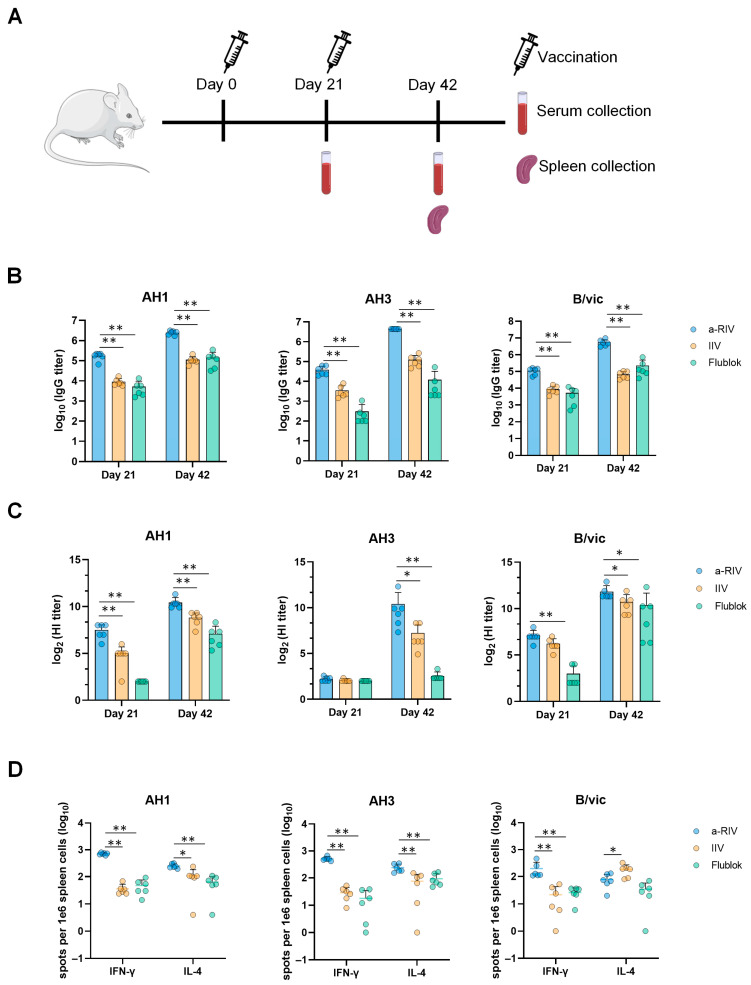
Comparison of humoral and cellular immune responses induced by a-RIV, IIV, and Flublok. (**A**) Immunization schedule: mice were immunized on days 0 and 21. Sera were collected on day 21 and day 42, and spleens were harvested on day 42 (n = 6). (**B**) Antigen-specific IgG titers against AH1, AH3, and B/vic were measured in sera collected on days 21 and 42 by ELISA. (**C**) HI titers against AH1, AH3, and B/vic were measured using standard antigens. (**D**) Frequencies of IFN-γ– and IL-4–secreting splenocytes were determined by ELISpot assay on day 42 after stimulation with peptide pools derived from AH1, AH3, and B/vic antigens. Data were analyzed using unpaired *t*-tests; * *p* ≤ 0.05, ** *p* ≤ 0.01; Non-significant difference is not shown.

**Figure 3:**
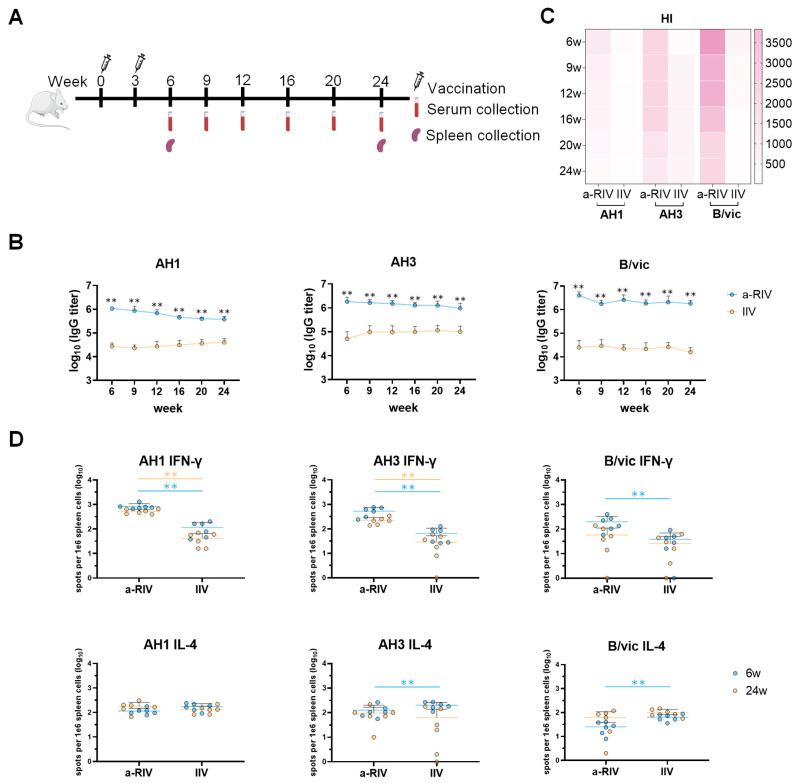
Durable humoral and cellular immune responses induced by Vaccination. (**A**) Immunization and sampling schedule: mice were immunized with a-RIV or IIV at week 0 and week 3 (n = 12). Sera were collected at weeks 6, 9, 12, 16, 20, and 24. Splenocytes were collected from 6 mice per group at weeks 6 and 24. (**B**) Antigen-specific IgG titers against AH1, AH3, and B/vic were measured at each indicated time point by ELISA. (**C**) HI titers were determined from serum samples collected at all time points. (**D**) The ELISpot assay was performed to quantify IFN-γ– (top) and IL-4– (bottom) secreting splenocytes at weeks 6 (blue) and 24 (yellow) following immunization with a-RIV or IIV. An unpaired *t*-test was used for statistical analysis. Significance is indicated as follows: ** *p* ≤ 0.01; Non-significant difference is not shown.

**Figure 4:**
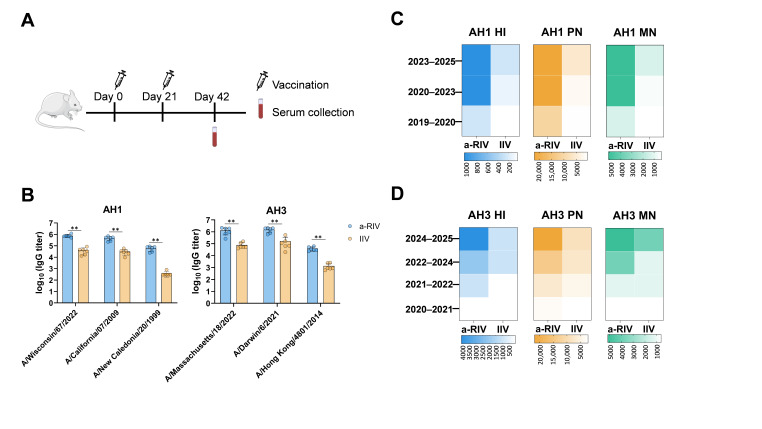
Neutralization of historical AH1 and AH3 viruses. (**A**) Immunization schedule: mice were immunized with a-RIV and IIV on days 0 and 21, and terminal sera were collected on day 42 (n = 6). (**B**) Antigen-specific IgG titers were measured by ELISA using soluble HA proteins from three representative AH1 strains (left) and three representative AH3 strains (right) as coating antigens. (**C**) Functional antibody responses to AH1 were evaluated using 2023–2025, 2020–2023, and 2019–2020 vaccine strains. HI titers (blue), PN titers (yellow), and MN titers (green) were measured using corresponding standard antigens, pseudoviruses, and viruses. (**D**) Functional antibody responses to AH3 were measured using 2024–2025, 2022–2024, 2021–2022, and 2020–2021 vaccine strains. HI titers (blue), PN titers (yellow), and MN titers (green) were determined accordingly. Unpaired *t*-tests were performed for group comparisons. Significance is indicated as follows: ** *p* ≤ 0.01; Non-significant difference is not shown.

Serum samples collected from immunized animals were analyzed for anti-HA total IgG endpoint titers using an indirect ELISA. Briefly, 96 well Nunc MaxiSorp plates (Thermo Fisher Scientific, Waltham, MA, USA) were coated with vaccine-matched recombinant HA proteins from Sino Biological corresponding to AH1 (catalog #40940-V08H7), AH3 (catalog #40992-V08H), and B/Victoria (catalog #40862-V08H) for experiments shown in Figure 1, Figure 2 and Figure 3. Each antigen was applied at 50 ng per well in 50 μL PBS and incubated at 2–8 °C for at least 12 h. For the cross-reactivity analysis (Figure 4), additional heterologous HA proteins were coated under identical conditions. These included AH1 antigens (catalog #11085-V08B and #11683-V08H) and AH3 antigens (catalog #40868-V08B and #40555-V08B), also obtained from Sino Biological. Following coating, plates were washed twice with PBS containing 0.05% Tween-20 (PBST) and blocked with 200 μL/well of Blocker Casein in PBS (Thermo Fisher Scientific) for 1 h at room temperature (RT). After two additional washes with PBST, serially diluted serum samples were added and incubated for 1 h at RT. Plates were then incubated with HRP-conjugated goat anti-mouse IgG (1:5000 dilution; Abcam, Cambridge, UK) for 1 h at RT, followed by six washes with PBST. TMB substrate (InnoReagents, Huzhou, China) was added and incubated for 10 min at RT, after which the reaction was stopped by the addition of 1 M HCl. Absorbance was measured at 450 nm with a reference wavelength of 620 nm. The endpoint titer was defined as the reciprocal of the highest serum dilution yielding an optical density above the established cutoff. Pre-immune sera from the same animals were used as baseline negative control.

### 2.7. Hemagglutination Inhibition (HI) Assay

HI titers were determined using standardized influenza virus antigens obtained from the National Institute for Biological Standards and Control (NIBSC, Potters Bar, Hertfordshire, UK). For Figure 1, Figure 2 and Figure 3, the following standard reagents were used: 22/320 (A/H1N1), 23/220 (A/H3N2), and 21/316 (B/Victoria). For the cross-reactivity analysis shown in Figure 4, l antigens were used: 22/320, 22/100, and 18/238 for AH1; 23/220, 21/314, 21/100, and 19/316 for AH3 Before testing, immune sera were treated with receptor destroying enzyme (RDE II; Denka Seiken,  Tokyo, Japan) according to the manufacturer’s instructions. Treated sera were serially twofold diluted in PBS (Gibco), starting at a 1:10 dilution, and incubated with four hemagglutination units (HAU) of influenza antigen for 1 h at room temperature. Subsequently, a 1% suspension of chicken RBCs was added to each well. For A/H3N2 strains, guinea pig RBCs were used instead. Plates were incubated for 30–60 min at RT. The HI titer was defined as the highest serum dilution that completely inhibited hemagglutination, as determined by visual inspection of the last well showing a distinct RBC button without agglutination.

### 2.8. Pseudovirus Neutralizing Assay (PN)

Pseudoviruses encoding influenza HA, NA, and firefly luciferase were provided by the National Institutes for Food and Drug Control (NIFDC, Beijing, China). Serum samples were heat inactivated at 56 °C for 30 min and then serially diluted in 3-fold steps using DMEM containing 2% FBS. The diluted sera were mixed with a fixed amount of pseudovirus and incubated at 37 °C for 1 h. Following incubation, 100 μL of the serum–virus mixture was added to 96-well plates pre-seeded with 2 × 10^4^ Huh-7 cells per well. Cells were then incubated at 37 °C in a humidified atmosphere containing 5% CO_2_ for 24 h. Finally, luciferase activity was measured to assess infection levels. The 50% pseudovirus neutralization titer was calculated using the Reed–Muench method and expressed as the reciprocal of the serum dilution that inhibited 50% of the luciferase signal. Pre-immune sera from the same animals were used as baseline negative control.

### 2.9. Microneutralization Assay (MN)

Heat inactivated serum samples were serially diluted in DMEM containing 1% BSA. A volume of 50 μL of each serum dilution was mixed with an equal volume of influenza virus containing 100 TCID_50_, and the mixtures were incubated in 96-well plates at 37 °C for 1 h. Subsequently, 1.5 × 10^4^ MDCK cells were added to each well, and plates were incubated at 37 °C with 5% CO_2_ for 18–22 h. After incubation, the cells were washed with PBS and fixed with cold acetone (pre-chilled at 4 °C) at room temperature for 10 min. Following removal of the fixative, 100 μL of mouse anti-influenza nucleoprotein monoclonal antibody (1:4000 dilution) was added to each well and incubated at room temperature for 1 h. After PBS washes, 100 μL of HRP-conjugated goat anti-mouse IgG (1:4000 dilution) was added and incubated for another hour at room temperature. Plates were then washed five times with PBS, and 100 μL of substrate solution was added to each well and incubated for 10 min for color development. The enzymatic reaction was terminated by adding 1 M sulfuric acid, and absorbance was measured at 450 nm with a reference wavelength of 620 nm using a microplate reader and the neutralizing antibody titer was calculated according to previously described methods [18]. Pre-immune sera from the same animals were used as baseline negative control.

### 2.10. Enzyme-Linked Immunospot Assay (ELISpot)

Following euthanasia, spleens were aseptically harvested from mice and mechanically dissociated to generate single cell suspensions. The suspensions were passed through a 70 μm cell strainer to remove debris. Red blood cells were lysed using in-house prepared Ammonium-Chloride-Potassium lysis buffer, and viable splenocytes were counted before the assay. The ELISpot assay was performed using the Mouse IFN-γ/IL-4 ELISpotPRO kit (Mabtech, Nacka, Sweden) according to the manufacturer’s instructions.

Briefly, pre-coated 96-well ELISpot plates were washed with PBS and blocked with 100 μL of α-MEM medium (Gibco) supplemented with 10% fetal bovine serum for 1–2 h at room temperature. After blocking, 2.5 × 10^5^ splenocytes per well were stimulated in a total volume of 100 μL with peptide pools at a final concentration of 0.25 μg/mL, the cells were separately stimulated with AH1 HA-specific peptides (56 overlapping 20-mer peptides with 10 amino acid overlaps, GenScript), AH3 HA-specific peptides (55 × 20-mer, GenScript), or B/vic HA-specific peptides (54 × 20-mer, GenScript). Then cells were incubated for 40 h at 37 °C in a humidified 5% CO_2_ incubator. After incubation, plates were washed with PBS and sequentially incubated with biotinylated detection antibodies against IFN-γ and IL-4 (Mabtech) for 2 h at room temperature, followed by streptavidin–alkaline phosphatase conjugate (Mabtech) for 1 h. Spot development was performed using BCIP/NBT-plus substrate for 8–10 min, after which the reaction was stopped by rinsing with distilled water. Plates were air dried and analyzed using an automated ELISpot reader (AID, Straßberg, Germany) to quantify cytokine secreting cells.

### 2.11. Statistical Analysis

Statistical analyses were performed using GraphPad Prism software (version 9.5.1). Comparisons between two groups were conducted using the nonparametric Mann–Whitney test. A *p*-value of <0.05 was considered statistically significant. For clarity, non-significant (ns) comparisons were not shown.

## 3. Results

### 3.1. Construction and Characterization of the Recombinant HA_mem_-VLP Mix and HA_sec_-NP Mix Vaccine

Based on the WHO recommendations for the 2024–2025 Northern Hemisphere cell- or recombinant-based influenza vaccines, the codon-optimized full-length and ectodomain HA sequences of A/Wisconsin/67/2022 (AH1), A/Massachusetts/18/2022 (AH3), and B/Austria/1359417/2021 (B/vic) were cloned into transfer plasmids and co-transfected with the baculovirus backbone plasmid into Sf9 insect cells to generate recombinant baculoviruses. These baculoviruses were subsequently used to infect Sf9 cells for recombinant HA protein expression.

The AH1, AH3, and B/vic HA_mem_-VLPs were subsequently extracted and purified. Size-exclusion chromatography (SEC) results showed that the elution times for AH3, B/vic, and AH1 were 10.099, 10.779, and 10.846 min, respectively, indicating the formation of high-molecular-weight oligomers; solvent peaks appeared after sample peaks (Figure 1A and Figure S1A). AH1, AH3, and B/vic HA_mem_-VLPs were combined at a 1:1:1 ratio to generate HA_mem_-VLP mix. Negative-staining electron microscopy of the HA_mem_-VLP mix revealed rosette-shaped, high-order oligomers with particle sizes of approximately 40 nm (Figure 1B). Together, these results confirm successful expression and purification of the HA membrane proteins, which can self-assemble into VLPs through hydrophobic interactions within their transmembrane domains, leading to spontaneous oligomerization into rosette-like structures [19].

In parallel, secreted HA proteins were produced using the same insect cell–baculovirus expression system. These HA_sec_ antigens were conjugated to the mi3 nanoparticle scaffold via isopeptide bonds, generating HA_sec_-NPs (Figure 1C and Figure S1B). SEC results revealed that HA_sec_-NPs eluted as a single, distinct peak at ~10 mL, whereas unbound HA_sec_ proteins eluted later, beyond 15 mL. Furthermore, when the three subtype-specific HA_sec_-NPs were combined in equal proportions to generate HA_sec_-NP mix, negative-stain electron microscopy confirmed the formation of uniformly distributed nanoparticles with an average diameter of ~46 nm (Figure 1D).

To directly compare the immunogenicity of the two platforms, HA_sec_-NP mix and HA_mem_-VLP mix were each formulated with 1.5 μg of hemagglutinin per strain (4.5 μg total per dose) and administered to BALB/*c* mice with an AS01-like adjuvant. Immunization results showed that the HA_mem_-VLP mix elicited higher antibody titers than the HA_sec_-NP mix (Figure 1E), likely due to the induction of additional antibodies against the mi3 carrier by HA_sec_-NPs (Figure S2). Based on these findings, subsequent vaccine formulations in this study were prepared using membrane-anchored HA, designated as adjuvanted-RIV (short for a-RIV).

### 3.2. The a-RIV Induced Stronger Humoral and Cellular Immune Responses than IIV and Flublok

To compare the humoral and cellular immune responses induced by a-RIV with those elicited by commercial influenza vaccines, BALB/*c* mice were immunized intramuscularly on days 0 and 21 with either a-RIV, IIV (2024–2025 season; Jiangsu Zhonghui Yuantong Biotech Co., Ltd. (Taizhou, China).; lot # A20240506), or recombinant influenza vaccine-Flublok (2024–2025 season, Sanofi, Paris, France, lot # TFAA2402). The doses for IIV and Flublok were set at 1/10 of the human dose [20], corresponding to 1.5 μg HA per subtype for IIV and 4.5 μg per subtype for Flublok, as defined by hemagglutinin content rather than protein quantity. a-RIV was administered at 1.5 μg HA per subtype. Blood samples were collected on days 21 and 42 for humoral response analysis, and spleens were harvested on day 42 for cellular immunity assessment (Figure 2A).

As shown in Figure 2B, ELISA results indicated that a-RIV induced significantly higher antigen-specific IgG titers against all three subtypes (AH1, AH3, and B/vic) compared to both IIV and Flublok. Consistently, hemagglutination inhibition assays (Figure 2C) demonstrated significantly higher HI titers in the a-RIV group, particularly against AH1 after the primary dose and against all three subtypes post-boost. Notably, IIV elicited higher functional antibody titers than Flublok after the second dose. Furthermore, under identical immunization conditions, we compared the immunogenicity of a-RIV formulated with an AS01-like adjuvant, an MF59-like adjuvant, and without adjuvant. As shown in Figure S3, the AS01-like formulation elicited significantly higher IgG titers, HI titers, and frequencies of IFN-γ–secreting splenocytes (as measured by ELISpot) compared to both the MF59-like adjuvant and unadjuvanted groups. Notably, the IgG and HI titers induced by the unadjuvanted a-RIV were comparable to those elicited by Flublok, with no statistically significant differences (Figure S4A,B). These results demonstrate that a-RIV elicits more robust humoral immune responses compared to both IIV and Flublok, while IIV is also more immunogenic than Flublok.

Given its role in viral clearance and long-term protection, T cell-mediated immunity is a key determinant of influenza vaccine efficacy [21,22]. ELISpot assays were used to quantify splenic lymphocytes secreting IFN-γ and IL-4 following stimulation with peptide pools specific for AH1, AH3, and B/vic. Although the IL-4 response to B/vic was comparable between a-RIV and Flublok and slightly higher with IIV, a-RIV elicited markedly enhanced IFN-γ and IL-4 responses against all other antigens relative to both IIV and Flublok (Figure 2D). Collectively, these findings demonstrate that a-RIV elicited stronger humoral and cellular immune responses than IIV and Flublok. In light of the superior immunogenicity of IIV over Flublok observed in several assessments, further investigations focused exclusively on the comparative evaluation of a-RIV and IIV.

### 3.3. Robust and Durable Humoral and Cellular Immune Responses Were Elicited by the a-RIV

The durability of immune responses induced by a-RIV and IIV was assessed over 24 weeks. As illustrated in Figure 3A, 24 female BALB/*c* mice were divided into two groups (n = 12) and immunized with either a-RIV or IIV at weeks 0 and 3. At week 6, six mice from each group were sacrificed for blood and spleen collection. The remaining six mice in each group were monitored longitudinally, with blood sampling at weeks 9, 12, 16, 20, and 24, and spleens collected at week 24 for ELISpot analysis.

As shown in Figure 3B, IgG titers elicited by a-RIV remained robust and relatively stable over time, showing only a minor decline within one logarithmic scale. Despite a slight decrease, antibody titers in the a-RIV group consistently exceeded those induced by IIV at all assessed time points. HI titers in the a-RIV group remained elevated for at least six months post-vaccination and were consistently higher than those observed in the IIV group at corresponding time points (Figure 3C). These results indicate that a-RIV elicited sustained and robust humoral immune responses throughout the 24-week observation period, with consistently higher levels compared to IIV.

Regarding the durability of T cell responses, ELISpot analyses at weeks 6 and 24 revealed that while IFN-γ–secreting cells exhibited only a slight decline over time, a-RIV maintained significantly higher levels compared to controls. a-RIV induced significantly stronger IFN-γ responses to all three subtypes at week 6, and this advantage persisted for influenza A subtypes at week 24. IL-4 responses remained stable or even slightly increased at week 24, with both a-RIV and IIV maintaining similar levels of IL-4–secreting cells. Overall, a-RIV induced more robust and durable humoral and Th-1 cellular immune responses than IIV, with sustained effects detectable at six months post-immunization.

### 3.4. The a-RIV Elicits Broadly Cross-Reactive Antibodies Against Heterologous Influenza Virus Strains

To evaluate the breadth of protection conferred by a-RIV, antibody responses to both homologous and heterologous influenza virus strains were assessed. Following the standard immunization schedule (Figure 4A), BALB/*c* mice received two doses of either a-RIV or IIV (1.5 μg HA per subtype) at a 3-week interval. Sera collected at week 6 were analyzed by ELISA, HI assay, pseudovirus neutralization, and microneutralization assays.

Due to the faster antigenic evolution of influenza A viruses compared to influenza B viruses [23,24], and the fact that WHO-recommended B/vic vaccine strains remained unchanged from 2022 to 2026, cross-reactivity analysis focused on influenza A subtypes. The a-RIV was formulated using the WHO-recommended strains for the 2024–2025 influenza season, while IIV was a commercially available inactivated vaccine for the same season.

ELISA showed that a-RIV elicited significantly higher IgG titers against both homologous (A/Wisconsin/67/2022 and A/Massachusetts/18/2022) and multiple heterologous strains compared to IIV (Figure 4B). In general, vaccine-induced immune responses were weaker against earlier-isolated strains. For AH1, strains from the 2019–2020, 2020–2023, and 2023–2025 seasons were used to evaluate HI titers, pseudovirus neutralization, and microneutralization (Figure 4C). For AH3, representative strains from the 2020–2021, 2021–2022, 2022–2024, and 2024–2025 seasons were tested (Figure 4D). In all assays, a-RIV elicited higher functional antibody titers across strains and seasons, indicating cross-protection over at least the past five years. These results demonstrate that a-RIV possesses superior breadth of antibody responses compared to IIV.

## 4. Discussion

In this study, we first generated HA_mem_-VLPs and HA_sec_-NPs using the baculovirus–insect cell expression system, with HA sequences based on the WHO-recommended strains for the 2024–2025 influenza season. The morphology and nanoparticle size distribution of HA_mem_-VLPs and HA_sec_-NPs were consistent with previous reports [25,26], confirming the successful assembly of these particles into virus-like particles and nanoparticles. Compared with the HA_sec_-NP mix, the HA_mem_-VLP mix induced stronger antibody responses in mice, which may be attributed to the fact that HA_mem_-VLP more closely resembles the native HA conformation on the influenza virus surface and that HA_mem_-VLP, unlike HA_sec_-NP, does not contain the mi3 nanoparticle carrier, thereby focusing the immune response exclusively on HA. Therefore, we focused subsequent studies on the immunogenicity of the adjuvanted HA_mem_-VLP mix vaccine, which we designated as a-RIV.

Immunogenicity evaluation in mice revealed that the a-RIV elicited significantly higher HA-specific binding antibody titers, HI titers, and T cell responses compared with both the licensed inactivated influenza vaccine and the recombinant protein vaccine Flublok. Notably, the a-RIV demonstrated enhanced durability and breadth of immune responses, maintaining elevated antibody and T cell levels for at least six months post-immunization—substantially outperforming IIV in this regard.

The a-RIV vaccine induced strong antibody responses not only against the homologous vaccine strains but also against multiple heterologous A/H1N1 and A/H3N2 strains from 2019 to 2025. ELISA results showed sustained high IgG binding titers across these drifted strains, while responses from IIV declined for earlier isolates. This was corroborated by consistently higher HI, pseudovirus neutralization, and microneutralization titers in the a-RIV group, confirming functional cross-reactivity. These findings suggest that a-RIV elicits antibodies capable of recognizing conserved epitopes across antigenically diverse strains. The improved performance of a-RIV likely results from multiple synergistic factors. Structurally, recombinant HA often exists as uncleaved HA0, preserving unique prefusion epitopes that can induce broadly cross-reactive and protective antibodies, particularly those targeting the conserved HA stem region. In addition, Hemagglutinin produced in the baculovirus–insect cell system carries mainly high-mannose, non-sialylated N-glycans, whereas HA expressed in human cells contains complex, branched, and sialylated glycans. These structural differences can influence antigenic properties by reducing glycan shielding and thereby exposing conserved epitopes, including those in the HA stem region [5,27]. Although insect-type glycans differ from native human patterns, they generally maintain correct folding and trimeric conformation, and the reduced glycan masking may help broaden cross-reactive antibody responses. Therefore, the simplified glycosylation of Sf9-derived HA may actually favor the induction of broad protective immunity. Furthermore, the use of the baculovirus–insect cell recombinant protein platform provides significant advantages over traditional egg- or cell-based vaccines. Unlike these conventional approaches, the recombinant protein platform does not rely on live influenza virus. It can be rapidly initiated by cloning the wild-type HA gene, thereby shortening the manufacturing timeline to 2–3 months, which is critical for seasonal updates and pandemic preparedness. Moreover, recombinant HA avoids the adaptive mutations introduced during viral propagation in eggs or mammalian cells, ensuring sequence fidelity to circulating strains and reducing the risk of antigenic mismatch.

Beyond the expression platform, the superior immunogenicity of a-RIV may also be attributed to its adjuvant formulation. Previous studies have demonstrated that oil-in-water adjuvants such as MF59 and saponin-based adjuvants like Matrix-M™ can enhance both innate and adaptive immune responses by promoting antigen uptake, dendritic cell activation, and recruitment of inflammatory cells [28,29,30,31,32,33]. Matrix-M™, for example, has shown promising results in enhancing antibody responses and CD4^+^ T cell immunity in the context of NanoFlu—a novel adjuvanted recombinant influenza vaccine currently in late-stage clinical trials [34]. Likewise, the AS01 adjuvant system—comprising MPL, QS-21, and liposomes—has been widely validated in malaria, RSV, and herpes zoster vaccines and is known to promote robust germinal center responses, CD4^+^ T cell polyfunctionality, and CD8^+^ T cell cross-presentation [35,36,37,38]. Unlike MF59 and Matrix-M™, AS01 engages TLR4-dependent signaling via MPL and synergistic saponin-induced antigen presentation to trigger a potent Th1-polarized immune profile with strong polyfunctional CD4^+^ T-cell responses and elicit long-lasting T-cell and antibody immunity [35]. So the AS01-like adjuvant was explored for the next generation of flu vaccine prototypes requiring strong T-cell responses. Supplementary Figure S3 demonstrates that the AS01-like formulation elicited significantly higher IgG titers, HI titers, and IFN-γ–secreting splenocytes (as measured by ELISpot) than the MF59-like adjuvant or unadjuvanted controls.

The hemagglutination inhibition assay remains the standard immunological correlate of protection for influenza, with an HI titer ≥40 generally associated with ~50% protection [39]. However, increasing evidence underscores the critical role of T cell–mediated immunity in influenza protection. Antigen-specific CD4^+^ T cells contribute by secreting cytokines such as IFN-γ, which support CD8^+^ T cell activation and memory maintenance [21,40], and in experimental human infections, pre-existing CD4^+^ T cells have been associated with reduced viral shedding and milder disease [41]. CD8^+^ cytotoxic T lymphocytes (CTLs) eliminate virus-infected cells by perforin/granzyme B release and Fas-mediated apoptosis [42], and they also produce IFN-γ to suppress viral replication [43]. Thus, an optimal influenza vaccine should elicit both humoral and cellular immunity. In addition to the strong IFN-γ responses observed, our ELISpot data demonstrated significant secretion of IL-4, particularly in response to AH1 HA antigens. IL-4, a canonical Th2 cytokine, plays a central role in B cell activation, class-switch recombination (particularly to IgG1), and the formation of germinal centers. These processes are essential for the generation of long-lived plasma cells and high-affinity antibody responses. a-RIV induced a balanced Th1/Th2 response (co-induction of IFN-γ and IL-4), which is advantageous for combined cellular and humoral immunity, despite eliciting IL-4 levels similar to non-adjuvanted IIV or Flublok.

To the best of our knowledge, this study represents one of the first direct comparisons between a membrane-anchored trivalent recombinant influenza virus-like particle (RIV) vaccine and a secreted HA–nanoparticle (HA_sec_-NP) vaccine, both produced via the baculovirus–insect cell expression system. This head-to-head comparison helps elucidate the relative immunogenicity of membrane-bound HA presented in VLP form versus scaffolded HA on the mi3 nanoparticle platform. In addition, this study includes a novel application of an AS01-like adjuvant in combination with a Flublok-like recombinant HA vaccine. While Flublok has been previously evaluated without adjuvants, the incorporation of an AS01-like adjuvant—known for its capacity to elicit potent Th1-biased cellular responses—has not been extensively explored in this context. Furthermore, we believe this work provides valuable comparative data by evaluating an adjuvanted trivalent recombinant HA VLP vaccine (a-RIV) alongside two licensed influenza vaccines: the inactivated influenza vaccine (IIV) and non-adjuvanted recombinant Flublok. Conducted under controlled conditions in a murine model, these comparisons offer new insights into the potential immunological advantages and durability of the a-RIV platform.

## 5. Conclusions

In summary, our results demonstrate that the a-RIV, a trivalent adjuvanted recombinant influenza virus-like particle vaccine, induces robust, durable, and broadly reactive humoral and cellular immune responses in mice. These results provide a strong rationale for advancing a-RIV toward further preclinical development and clinical testing as a next-generation influenza vaccine candidate.

Despite these promising findings, this study has limitations. Our evaluation was limited to mouse models, and no viral challenge experiments were conducted to directly assess protective efficacy. Future work should include homologous and heterologous challenge studies in animal models and clinical evaluation to validate the protective potential of a-RIV.

## Data Availability

Data will be made available on request.

## Supplementary Materials

The following supporting information can be downloaded at: https://www.mdpi.com/article/10.3390/vaccines13111162/s1, Figure S1. Characterization of HA_mem_-VLP and HA_sec_-NP by SDS-PAGE; Figure S2. Characterization of mi3 scaffold-specific IgG titers. Mi3; Figure S3. Comparison of the immunogenicity between unadjuvanted RIV and adjuvanted RIV with MF59-like or AS01-like adjuvant; Figure S4. Comparison of the immunogenicity between unadjuvanted RIV and Flublok.

## Abbreviations

The following abbreviations are used in this manuscript:

WHO	World Health Organization
a-RIV	adjuvanted trivalent recombinant influenza virus-like particle vaccine
RIV	trivalent recombinant influenza virus-like particle vaccine
IIV	inactivated influenza vaccine
HA	hemagglutinin
AH1	A/H1N1
AH3	A/H3N2
B/vic	B/Victoria
LRTI	lower respiratory tract infection
VE	vaccine effectiveness
CDC	Centers for Disease Control and Prevention
NA	neuraminidase
DMEM	Dulbecco’s Modified Eagle Medium
FBS	fetal bovine serum
MOI	multiplicity of infection
PB	phosphate buffer
IACUC	Institutional Animal Care and Use Committee
ELISA	enzyme-linked immunosorbent assay
RT	room temperature
HI	hemagglutination inhibition
PN	pseudovirus neutralizing assay
MN	microneutralization assay
ELISpot	enzyme-linked immunospot assay
HA_mem_	HA membrane protein
HA_sec_	HA secretory protein
SEC	size-exclusion chromatography
CTL	cytotoxic T lymphocyte
DOPC	1,2-dioleoyl-sn-glycero-3-phosphocholine
MPL	monophosphoryl lipid A
Tween-20	polysorbate 20
NIFDC	National Institutes for Food and Drug Control
HAU	hemagglutination unit
HA_mem_-VLP	HA membrane protein virus-like particle
HA_sec_-NP	HA_sec_ nanoparticle
CHT	ceramic hydroxyapatite
