# Supplementary material for: Adjuvanted Recombinant Hemagglutinin Vaccine Provides Durable and Broad-Spectrum Immunogenicity in Mice
Source: Vaccines (Basel). 2025 Nov 14;13(11):1162. doi: 10.3390/vaccines13111162 (PMC12656808; doi:10.3390/vaccines13111162)
Supplement: Supplementary file 1 [file vaccines-13-01162-s001.zip › vaccines-3930062-supplementary.pdf]

Supplemental Figures

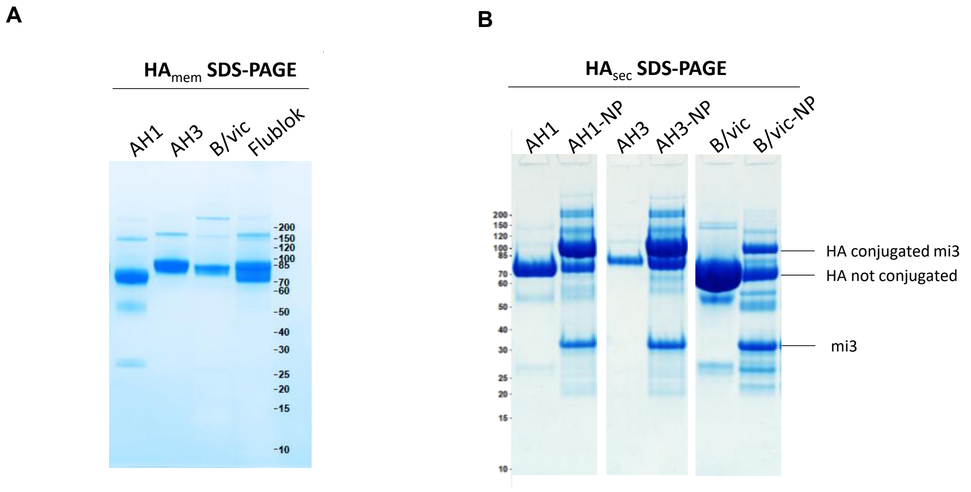

Figure S1. Characterization of HAmem-VLP and HAsec-NP by SDS-PAGE. (**A**) Reducing SDS-PAGE analysis of HAmem-VLP and Flublok (**B**) Reducing SDS-PAGE analysis of the soluble HAsec trimers or HAsec-NP that HAsec trimers conjugated the scaffold mi3.

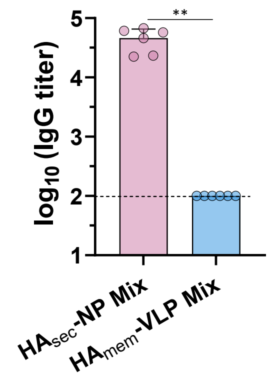

Figure S2. Characterization of mi3 scaffold-specific IgG titers. Mi3 scaffold-specific IgG titers of HAsec-NP mix and HAmem-VLP mix were measured in serum samples after two immunizations. Statistical significance was determined using an unpaired *t*-test (** $p \leqslant 0.01$).

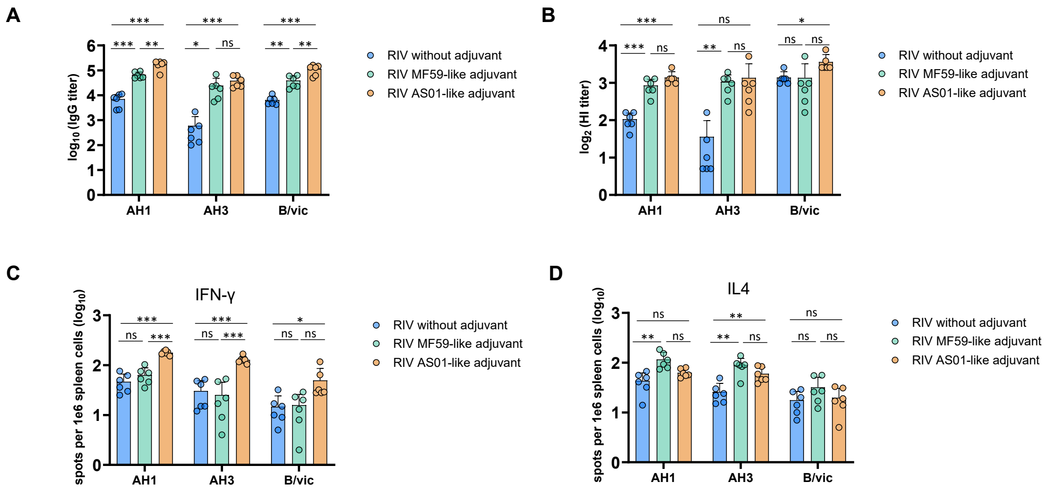

Figure S3. Comparison of the immunogenicity between unadjuvanted RIV and adjuvanted RIV with MF59-like or AS01-like adjuvant.
(**A**) Antigen-specific IgG titers and (**B**) HI titers were measured in sera from mice immunized with RIV unadjuvanted or adjuvanted to compare humoral immune responses. (**C**) Frequencies of IFN-γ– and (**D**) IL-4–secreting splenocytes were determined by ELISpot assay on day 42 after stimulation with peptide pools derived from AH1, AH3, and B/vic peptide. Data were analyzed using un-paired *t*-tests; * $p \leqslant 0.05$, ** $p \leqslant 0.01$, *** $p \leqslant 0.001$.

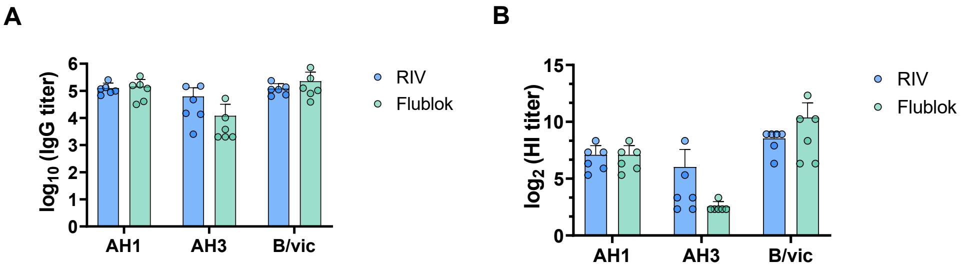

Figure S4. Comparison of the immunogenicity between unadjuvanted RIV and Flublok. (**A**) Antigen-specific IgG titers and (**B**) HI titers were measured in sera from mice immunized with RIV or Flublok to compare humoral immune responses. Data were analyzed using unpaired *t*-tests, ns is not shown.
